# Therapeutic Alliance in a Single Versus Group Rehabilitative Setting After Breast Cancer Surgery: Psychological Profile and Performance Rehabilitation

**DOI:** 10.1089/biores.2019.0011

**Published:** 2019-07-03

**Authors:** Teresa Paolucci, Andrea Bernetti, Marco Paoloni, Serena V. Capobianco, Arianna V. Bai, Carlo Lai, Laura Pierro, Monica Rotundi, Carlo Damiani, Valter Santilli, Francesco Agostini, Massimiliano Mangone

**Affiliations:** ^1^ Complex Unit of Physical Medicine and Rehabilitation, University Hospital Umberto I, Rome, Italy.; ^2^ Department of Anatomical and Histological Sciences, Legal Medicine and Orthopedics, Sapienza University of Rome, Rome, Italy.; ^3^ Department of Dynamic and Clinical Psychology, Sapienza University of Rome, Rome, Italy.; ^4^ Department of Neurorehabilitation, IRCCS San Raffaele Pisana, Rome, Italy.

**Keywords:** breast cancer, exercise, MMPI-2 scale, quality of life, rehabilitation, setting

## Abstract

The survival rate of women after breast cancer has improved significantly worldwide. More attention should be paid to the rehabilitation intervention after surgery. Cancer rehabilitation helps breast cancer survivors maintain the highest possible physical, social, psychological, and vocational function in the limits that are imposed by the cancer and its treatments. The aim of our research was to determine the rehabilitative setting that promotes greater efficacy of the rehabilitation. A double-blind, randomized controlled trial with 45 patients enrolled was conducted. All participants were randomized into two groups: single rehabilitative training (*N* = 22) and group rehabilitative training (*N* = 23). Outcomes were assessed for each group before treatment (T0), after first 6 weeks of rehabilitative treatment (T1), and after 3 months (T2). All patients underwent the same rehabilitation treatment, but the setting differed between single and group rehabilitative training, which included four to five patients each and evaluated using Minnesota Multiphasic Personality Inventory (MMPI-2), Working Alliance Inventory Patient form (WAIP), Disabilities of Arm, Shoulder and Hand Questionnaire (DASH), and visual analog scale (VAS). Two patients dropped out in the single treatment group. In the within-group analysis at the three evaluation times, on the VAS, a significant reduction in pain was reported and maintained at the follow-up, as was observed for the DASH and WAIP scales. In the between-group analysis WAIP and Bond scale scores differed significantly in favor of the single treatment. In the group treatment, the Psychopathic Deviate, Masculine/Feminine, and Social Discomfort scales of the MMPI-2 correlated with WAIP Tot at T1. There was an association between the Correction, Hysteria, Paranoid, and Schizophrenia MMPI-2 scales and Δ VAS T0T1 in the total sample. Proposing the same rehabilitative intervention in both breast cancer groups, our results showed significant reduction in pain and good functional recovery of the upper limb, which did not depend on the setting (single or group). However, with single rehabilitation treatment, patients developed a better therapeutic alliance and experienced a more comfortable environment.

## Introduction

The survival rate of women after breast cancer has improved significantly worldwide.^1^ Thus, more attention should be paid to the rehabilitation intervention after surgery, which is commonly associated with such disorders as shoulder dysfunction, postmastectomy syndrome, chemotherapy-induced peripheral neuropathy, axillary cording, and lymphedema. Patients who participate in exercise before, during, and after treatment for breast cancer are more likely to return to work. Moreover, the social aspect is important for breast cancer survivors, and a woman's need to care for children, perceived body image, and existential well-being can also affect her return to work.^2^ Home-based multidimensional survivorship programs are effective for breast cancer survivors with regard to quality of life and functional improvement.^3–5^

Cancer rehabilitation helps breast cancer survivors and others attain and maintain the highest possible physical, social, psychological, and vocational function in the limits that are imposed by the cancer and its treatments.^6^ Moreover, immediate breast reconstruction has been increasingly incorporated into breast cancer treatment, especially for its psychological benefits,^7,8^ and facilitates the recovery of upper limb function and posture during rehabilitation. Yet, no studies have examined which treatment, between individual and group settings, is better in breast cancer after surgery.

Robertson and Harding made such an attempt, considering other diseases, such as back pain, and they concluded that rehabilitation in a group format results in equivalent clinical outcomes as a similar therapy in an individual setting for the treatment of back pain. However, their evidence is insufficient to draw similar conclusions in other populations and areas of rehabilitation.^9^ Although rehabilitation is a fundamental element after breast cancer surgery, the qualitative aspects of the therapeutic alliance between the patient and physiotherapist have not been studied extensively.

The potential benefits of improving the therapeutic alliance include better adherence to exercise.^10^ Therapeutic alliance refers to the relational processes in rehabilitative treatment that can act in combination with or independent of a specific setting (individualized or group treatment). The therapeutic alliance between the patient and physiotherapist can depend on the rehabilitation setting and the personality of each participant.

Certain aspects of personality disorders can affect the therapeutic alliance, such as impairments in interpersonal relationships in paranoid, schizoid, and schizotypal personality disorders and the tendency to push one's limits in borderline personality disorder.^11^ Similarly, this cluster of patients (paranoid, schizoid, and schizotypal) has difficulties establishing a working alliance, due to their refusal of relationships and the belief that other people are hostile and threatening.^12^ For breast cancer survivors, their psychological aspects and personality profiles are important, because they can influence the rehabilitation process and its success.

The styles with which survivors cope with cancer are predictive of their psychological symptoms, psychological well-being, and health-related quality of life but not cancer survival or recurrence.^9^ In addition, personality is associated with psychological and physical symptoms in cancer patients, in particular, a “*resilient*” attitude is linked to extraversion, agreeableness, and conscientiousness,^10^ and anxiety is directly related to coping, in which a low level of anxiety is associated with good problem-solving strategies, whereas emotion-focused coping is applied at medium to high levels of anxiety.^13^

Thus, the aim of our research was to determine the rehabilitative setting—a single or group setting—that is more suitable for a better therapeutic alliance and promotes greater efficacy of the rehabilitation with respect to the function and pain of the upper limb. As a secondary outcome, we examined the variables of the patient's personality profile that are associated with the therapeutic alliance.

## Materials and Methods

**FIG. 1.**
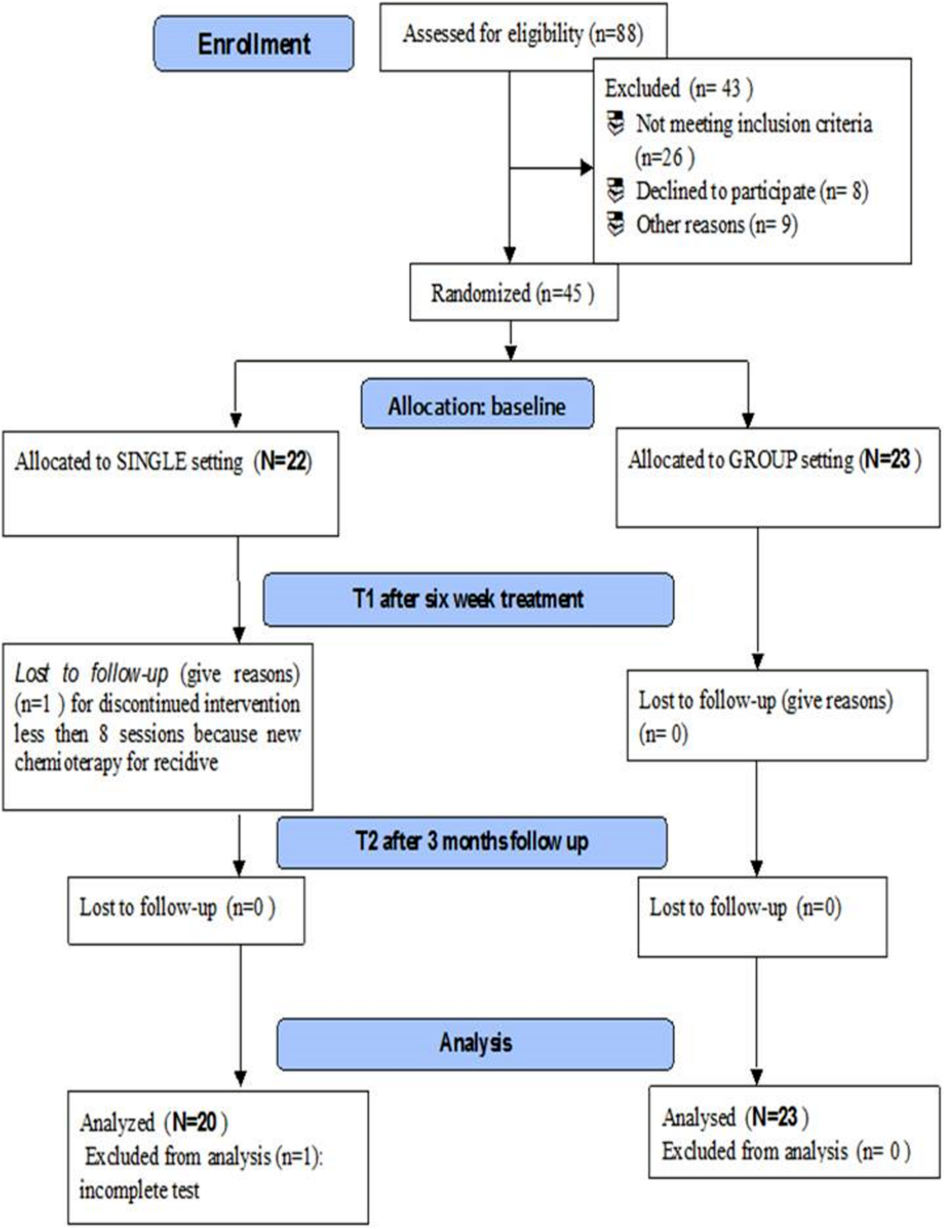
Flowchart.

This study was a double-blind, randomized controlled trial that took place from January 2016 to September 2018 at the Rehabilitation Outpatient Clinic of University Hospital Umberto I of Rome, Italy. A total of 88 women, after breast cancer surgery, were referred to a physiatric consultation by their oncologist; 45 patients enrolled, because they met the inclusion criteria and agreed to participate. All participants were randomized into two groups according to a computer-generated simple randomization list at a 1:1 ratio (software MATLAB R2007b^®^; The MathWorks, Inc.): single rehabilitative training (*N* = 22) and group rehabilitative training (*N* = 23) (Fig. 1, flowchart).

The inclusion criteria were as follows: total mastectomy that had been performed within 12 months before recruitment, age 18 to 60 years, body mass index (BMI) <30, and no cognitive dysfunctions (Mini-Mental State Examination >24).^14^ The exclusion criteria were as follows: presence of lymphangitis or mastitis, surgical complications of the intervention, neurological deficits and complications, significant shoulder joint problems before the intervention for breast cancer, previously diagnosed postural problems (such as scoliosis >10° Cobb angle), severe lymphedema and web axillary syndrome, and psychiatric or psychological problems in pharmacological treatment.

All participants signed informed consent forms after receiving detailed information on the study aims and procedures as per the Declaration of Helsinki. The rights of human subjects who were involved in the study were protected. The study protocol was approved by the Ethics Committee of University Hospital Umberto I—Sapienza University of Rome. The pharmacological therapeutic regimen must have been stable for at least 1 month before the patient began treatment. No new medications or other rehabilitation approaches were undertaken during the study.

The patient's baseline medical history, height, weight, and BMI were collected by the physiatrist of the rehabilitation center, and a clinical examination was performed. The shoulder joint range of motion on the operated side was evaluated by the physiatrist, and muscular strength was assessed with the Medical Research Council and Manual Muscle Testing scale.^15^

The patients, the physiatrist who enrolled them, and the researcher (psychologist or physiatrist) who administered the evaluation scales were blinded to the rehabilitative treatment.

This study protocol was developed in accordance with the Consolidated Standards of Reporting Trials (CONSORT) guidelines.^16^

Outcomes were assessed for each group before treatment (T0 = baseline), at the end of the first 6 weeks of rehabilitative treatment (T1 = 12 sessions, 2/week, 60 min for session), and after 3 months of follow-up (T2). The Minnesota Multiphasic Personality Inventory (MMPI-2) Profile^17^ was performed only at T0. To reduce the potential for bias, all patients were evaluated by the same blinded researcher at T0, T1, and T2.

### Evaluation scales

#### Psychological profile

The MMPI-2 is the most widely used personality tool, comprising 10 personality scales and 3 validity scales.^18^ The questionnaire is composed of 567 items with dual alternative response (True or False). We recorded the basic scales: Lie (L), Frequency (F), and Correction (K). The clinical scales are the basis for probing the most significant dimensions of personality: Hypochondriasis (HS), Depression (D), Hysteria (HY), Psychopathic Deviate (PD), Masculinity-femininity (MF), Paranoid (PA), Psychasthenia (PT), Schizophrenia (SC), Hypomania (MA), and Social Introversion (SI). The content scales that allow you to describe a different personality were considered as supplementary scales.

#### Therapeutic alliance

The Working Alliance Inventory Patient form (WAIP) consists of 36 items that are organized into 3 subscales as follows: Bond (positive emotional bond), Goal (agreement on treatment), and Task (therapeutic tasks), with 12 items each. Users respond to each item using a 7-point Likert scale, and scores range from 36 to 252 points.^19–21^

#### Disability of upper limb

The Disabilities of Arm, Shoulder and Hand Questionnaire (DASH)^22,23^ is a 30-item, self-reported questionnaire that is designed to measure physical functions and symptoms in musculoskeletal disorders of the upper limb. The items are related to the degree of difficulty in performing various functional activities due to arm, shoulder, or hand troubles (21 items); the severity of pain, activity-related pain, tingling, weakness, and stiffness (5 items); and the effects on social activities, work, and sleep and their psychological impact (4 items). A higher score (0–100) reflects greater disability.

#### Pain

The visual analog scale (VAS) is a simple, robust, sensitive, and reproducible instrument that enables the patients to express their pain intensity as numerical values on a line from 0 to 10 cm. Patients associated the severity of their upper limb pain with respect to the side of surgery to a continuous 10-cm line that was marked “no pain” on one end and “worst pain” on the other.^24^

### Rehabilitation treatment

All patients underwent the same rehabilitation treatment, but the setting differed between single and group rehabilitative training, which included four or five patients each. Three physiotherapists, who were experts in oncological rehabilitation, alternated randomly between single and group rehabilitation. The physiotherapists tailored the rehabilitation to the patients' functional problems (i.e., lymphedema, reduction in shoulder range of motion, postural alignment, and upper limb pain) and guided the motor rehabilitation. At the end of the rehabilitation, patients were invited to continue the exercises at home using a booklet that explained and illustrated the exercises that were to be followed progressively.

The rehabilitation treatment has generated good results as follows^25^:

(1) Diaphragmatic breathing and postural elements, such as alignment of the midline; (2) raising the arm, opening and closing hands; (3) stretching and releasing the arms and recovery of flexion; (4) turn the shoulders and rotation-anteropulsion-retropulsion; (5) abduction and adduction of the arms and isometric strengthening; (6) opening and closing the elbows; (7) run up a wall with upper limb for the recovery of flexion; (8) run up a wall with surgery upper limb by the side for the recovery of abduction; (9) bring the hand of the operated limb to the contralateral shoulder for the recovery of adduction; (10) rotate the arms for promoting rotation; (11) while standing, place the hands behind the back and take the hand of the operated limb with the healthy hand and slowly slide the hands along the spine upward to its possible and maintain the position for several seconds; (12) bar exercise for upper limb flexion, extension, and rotation; (13) Codman's pendulum (Patient with the trunk bent forward with the arm involved hanging, perpendicular to the ground, and the muscles of the arms and shoulders relaxed. The patient is asked to move his arm slowly, increasing the range of motion as tolerated); and (14) flex on the front wall to prevent deficit of the scapula.

**FIG. 2.**
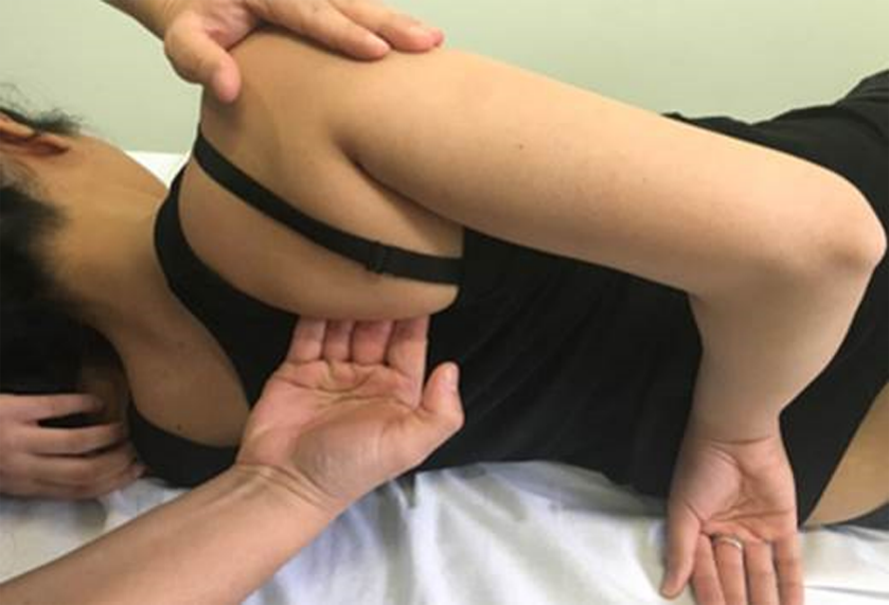
Soft tissue mobilization of scapula thoracic area.

**FIG. 3.**
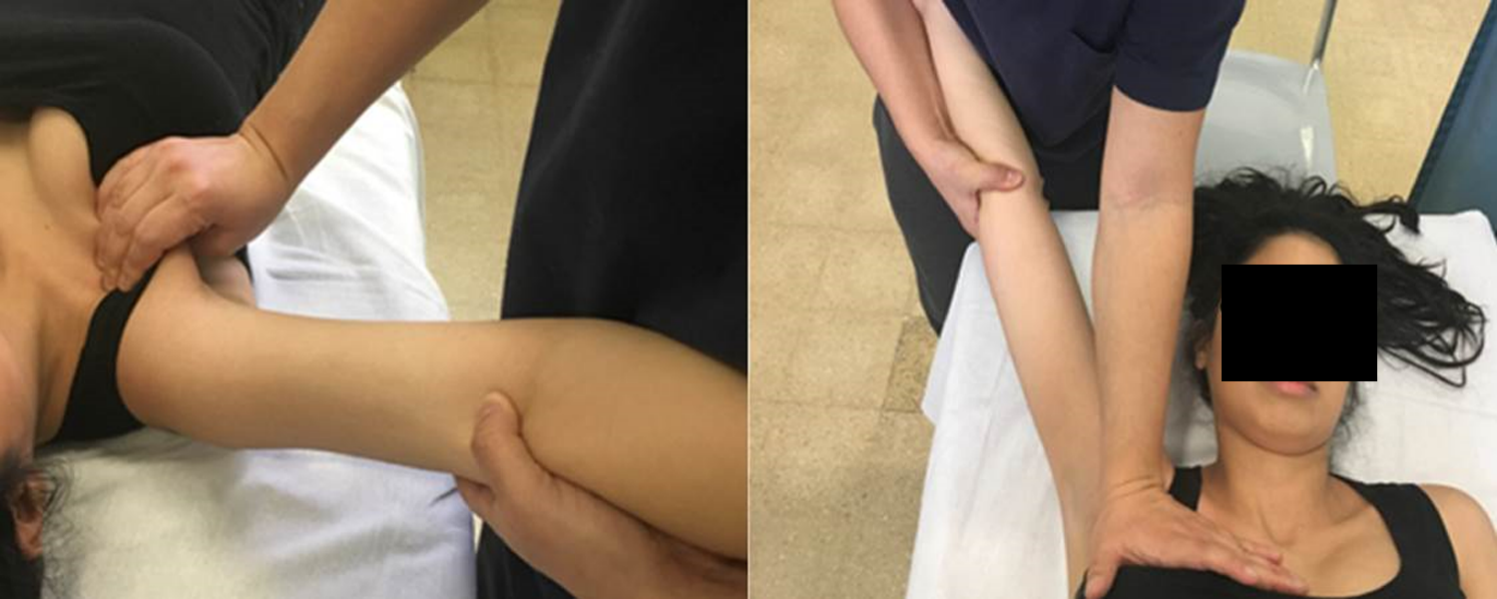
Stretching of pectoralis muscles.

All sessions started with at least 15–20 min of low-impact aerobics warm-up. All proposed exercises were repeated, starting from 10 repetitions for 3 times (adapting the increase in performance to the patient's compliance and resistance, progressing gradually during the sessions). In addition, three specific exercises were added to the protocol by the physiotherapist: mobilization of the thoracic scapula joint, cervical pumping, and lengthening of the pectoral muscles (Figs. 2 and 3). For patients who presented with mild-to-moderate lymphedema, additional lymph drainage sessions were held.

### Sample size calculation

The sample size was calculated using the VAS as the primary outcome with respect to rehabilitation; a power of analysis of 90% and alpha = 0.05 were considered. As a result, 16 patients were needed per group. Considering a 20% dropout, we aimed to enroll at least 20 patients/group to observe the minimal clinical difference in VAS scores: the primary end-point was defined as a difference of ∼2 points on the VAS between the 2 groups after treatment, assuming a standard deviation of 1.5 (using the online sample size calculator that was developed by DSS Research).^26^

### Statistical analyses

Mean and standard deviation were calculated for MMPI-2-computed variables. Values are expressed as median and interquartile range for continuous variables and as proportion for categorical variables, as appropriate. Related-samples Friedman's two-way analysis of variance by rank test was performed to assess the changes in scale scores in each group at the three time points. Independent-samples Mann–Whitney *U* test was used to compare scale scores between the two groups at each time point. Subsequently, pairwise comparison with Bonferroni correction was performed for each parameter.

At baseline, unpaired *t*-test was performed to determine the two groups of subjects who were matched for age and BMI. Analysis of variance was used to test the difference between groups (single vs. group) with regard to the personality profile in the MMPI-2 score. Pearson correlation was used to compare MMPI-2 subscales at T0 with WAIP tot at T1, ΔVAS T0T1, ΔVAS T0T2, ΔDASH T0T1, and ΔDASH T0T2 for the total sample, single treatment, and group treatment. All data analyses were performed using IBM SPSS for MACv.21 (IBM SPSS, Inc., Chicago, IL). The threshold of significance was set to a *p*-value of 0.05 for all tests.

## Results

During the 6-week treatment period, two patients dropped out in the single treatment group for discontinuing intervention with chemotherapy for recidive and other medical problems.

**FIG. 4.**
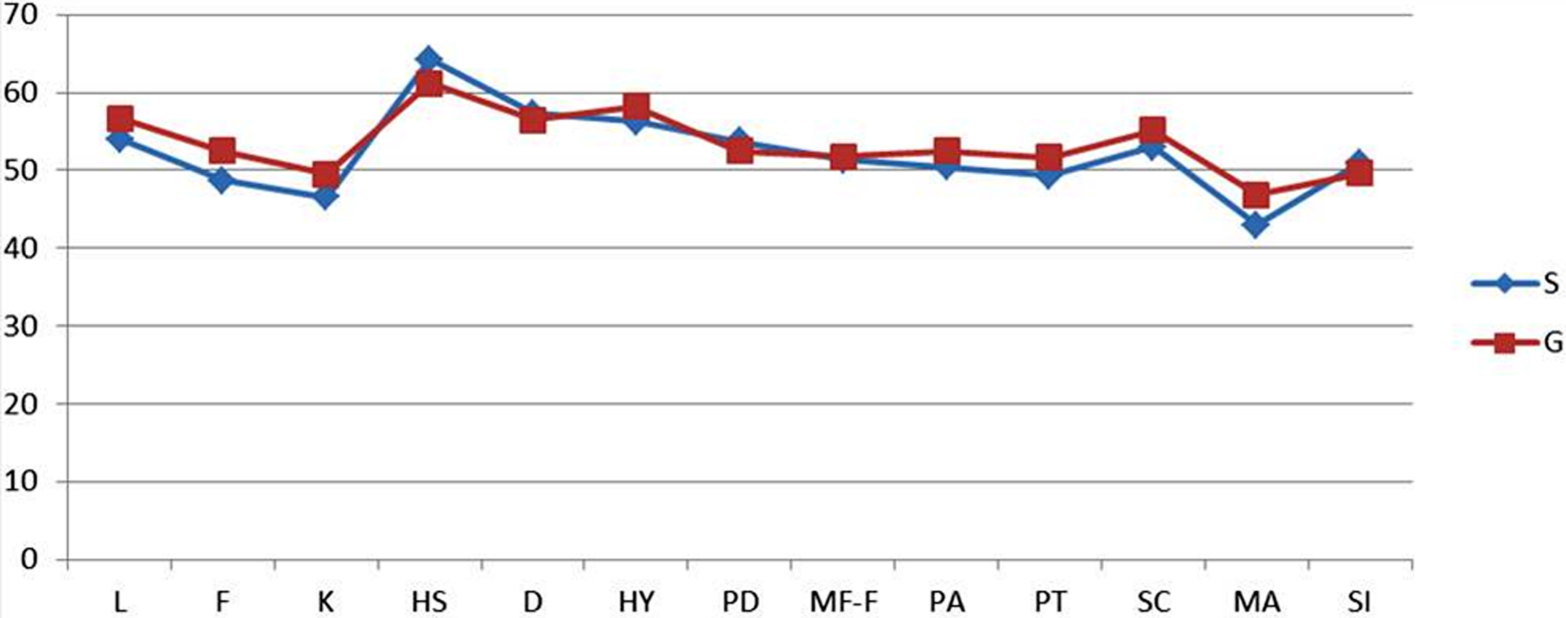
MMPI-2 profile in the two rehabilitative treatment groups (pt.). Lie (L), Frequency (F), Correction (K), Hypochondriasis (HS), Depression (D), Hysteria (HY), Psychopathic Deviate (PD), Masculinity-femininity (MF), Paranoid (PA), Psychasthenia (PT), Schizophrenia (SC), Hypomania (MA), and Social Introversion (SI). MMPI-2, Minnesota Multiphasic Personality Inventory.

Thus, the data for 43 patients—20 in personalized and 23 in group rehabilitation treatment—were analyzed (Fig. 4). The groups were matched for age, BMI, and time from surgery (months) (Table 1). No subject reported exacerbation of painful symptoms during or after the rehabilitative intervention.

**Table 1.** Baseline Characteristics of Both Groups

Clinical aspects	Group treatment	Single treatment	*p*
Patients, *n*	23	20	—
Age, mean ± SD (range)	52.6 ± 7.8 (60–41)	51 ± 9.6 (60–36)	0.68
BMI, mean ± SD (range)	24 ± 3.5 (30–20)	23.87 ± 4.4 (29–21)	0.98
Time from surgery [months]	5.36 ± 4.03	4.80 ± 4.85	1.58
Married/common-law spouse	80%	78%	—
Job	Working 63%	Working 68%	—
Not employed	Not employed 15%	Not employed 22%	
Retired from work	Retired 22%	Retired 10%	
High school or master's degree education (17 years of school)	30%	38%	—
Chemotherapy^a^	49%	43%	—
Radiotherapy^a^	58%	52%	—
Mild lymphedema	10%	8%	—

^a^ Before rehabilitation treatment.

BMI, body mass index; SD, standard deviation.

### Within-group analysis

In the within-group analysis at the three evaluation times, on the VAS, a significant reduction in pain was reported and maintained at the follow-up (*p* < 0.001), as was observed for the DASH (*p* < 0.001) and WAIP scales (*p* = 0.005 single; *p* = 0.048 group) (Table 2).

**Table 2.** Descriptive Values of the Scales in the Two Groups (Median—Minimum and Maximum) and Between Groups

Times	T0		T1		T2		*p*^a^ <0.05		*p*^b^ <0.05	
Rehabilitation	Single	Group	Single	Group	Single	Group	Single	Group	Between	Group
Scales	Median (min-max)								Δ T0T1	Δ T1T2
VAS	2.00 (0–4)	2.00 (0–7)	1.00 (0–2)	0.00 (0–4)	0.50 (0–2)	0.00 (0–2)	**<0.001**	**<0.001**	0.99	0.92
WAIP tot	206.00 (205–252)	208.00 (145–252)	236.00 (213–252)	225.50 (166–252)	240.00 (207–250)	214.00 (136–252)	**0.005**	0.048	**0.03**	**0.04**
Bond	20.00 (9–21)	21.00 (18–21)	21.00 (13–21)	21.00 (18–21)	21.00 (11–21)	21.00 (16–21)	0.42	0.156	**0.01**	**0.001**
Goal	35 (20–42)	36.00 (25–42)	34.50 (23–42)	40 (34–42)	34.50 (20–42)	40.00 (25–42)	0.20	**0.050**	0.35	0.85
Task	33.50 (15–35)	35.00 (22–35)	34.50 (15–38)	35.00 (23–38)	35.10 (15–38)	35.00 (24–38)	0.069	0.291	0.76	0.37
DASH	27.60 (1.7–62.5)	30.00 (0.8–68.3)	22.00 (0.00–52.50)	13.00 (0.0–49.2)	16.25 (0.00–50.0)	1.00 (0.0–46.7)	**<0.001**	**<0.001**	0.52	0.81

Post hoc analysis						
Times	T1–T2		T0–T2		T0–T1	
Rehabilitation setting	Single	Group	Single	Group	Single	Group
Scales						
VAS	1	1	**<0.001**	**<0.001**	**0.001**	**<0.001**
WAIP tot	**0.04**	—	1	—	0.031	—
Bond	**0.042**	—	0.536	—	0.157	—
Goal	—					
Task	—					
DASH	0.3134	0.113	**<0.001**	**0.001**	**0.006**	**0.001**

^a^ *p* = within group.

^b^ *p* = between group.

DASH, Disabilities of Arm, Shoulder, and Hand Questionnaire; T0, before treatment; T1, after first 6 weeks of rehabilitative treatment; T2, after 3 months; VAS, visual analog scale; WAIP, Working Alliance Inventory Patient form.

### Between-group analysis

In the between-group analysis (Table 2), no significant differences were seen on the VAS or DASH scale. In contrast, WAIP and Bond scale scores differed significantly in favor of the single treatment (ΔT0T1 WAIP *p* = 0.03; ΔT1T2 *p* = 0.04; ΔT0T1 Bond *p* = 0.01; ΔT1T2 Bond *p* = 0.00).

### MMPI-2 profile, rehabilitation, and therapeutic alliance: associated factors

The between-group analysis (single vs. group) did not show any differences in personality profile on the MMPI-2 at T0 [Wilks (28,10) = 0.8 (*p* = 0.696)].

In the total sample, by Pearson correlation, the Masculine/Feminine and Anxiety scales of the MMPI-2 correlated with WAIP Tot at T1 (*r* = 0.366 *p* = 0.022; *r* = 0.397 *p* = 0.012). Similarly, in the single treatment group, the Lie and Anxiety scales of MMPI-2 correlated with WAIP Tot at T1 (*r* = −0.605 [*p* = 0.013] and *r* = 0.589 [*p* = 0.016], respectively).

In the group treatment, the Psychopathic Deviate, Masculine/Feminine, and Social Discomfort scales of the MMPI-2 correlated with WAIP Tot at T1 (respectively, *r* = −0.4399 [*p* = 0.036]; *r* = −0.6575 [*p* = 0.001]; *r* = 0.4403 [*p* = 0.035]).

Furthermore, there was an association between the Correction, Hysteria, Paranoid, and Schizophrenia MMPI-2 scales and Δ VAS T0T1 in the total sample (*r* = −0.325 *p* = 0.047; *r* = −0.380 *p* = 0.018; *r* = −0.373 *p* = 0.021; and *r* = −0.382 *p* = 0.018, respectively). In the single treatment group, there was a negative correlation between Correction and Δ VAS T0T1 (*r* = −0.555 *p* = 0.026). In the group treatment, the Hypochondriasis, Hysteria, Paranoid, and Cynicism MMPI-2 scales correlated with Δ VAS T0T1 (*r* = −0.0438 *p* = 0.042; *r* = −0.462 *p* = 0.030; *r* = −0.474 *p* = 0.026; and *r* = 0.429 *p* = 0.046, respectively).

No link between MMPI-2 scales and Δ VAS T0T2, Δ DASH T0T1, and Δ DASH T0T2 was observed in the total sample or single and group treatments.

## Discussion

The main result of this study is that single and group rehabilitative treatments affect functional recovery of the upper limb and reduce pain. The proposed rehabilitative treatment with low aerobic impact, with a frequency of twice weekly, promoted good adherence to the treatment, with limited dropout (less than 20%), and the proposed exercises were well supported, even by those who were in chemotherapy or radiotherapy.

These data are encouraging, because although exercise is associated with numerous benefits in women with breast cancer, adherence to exercise training during cancer treatment is challenging.^27^ A patient's capacity to engage in the rehabilitative process varies over the course of cancer therapy and into survivorship. Perioperative attention generally focuses on managing premorbid impairments and normalizing shoulder function, but during chemotherapy and radiation therapy, symptom control, constructive coping, and role preservation might become more salient.^28^ Moreover, low-intensity exercise can assist in preventing cognitive dysfunction during or after chemotherapy in patients with breast cancer.^29^

Although single and group rehabilitative treatment had good efficacy, only the single setting promoted a better therapeutic alliance, based on the WAIP scale, perhaps because it allows greater individualization of the rehabilitation treatment and exclusive sharing of time and contents with the physiotherapist, to the detriment of a reduction in emotional sharing with peers. It is essential to attend to the patient as a person with unique experiences, perspectives, and attitudes and to modify the treatment, based on her priorities.^30^ As in our study, in single treatment, the physiotherapist has greater access to the patient.

The therapeutic relationship between the patient and physiotherapist is a central component of patient-centered care and is positively associated with better clinical physiotherapy outcomes. Four conditions were identified as being necessary for establishing a therapeutic relationship: present, receptive, genuine, and committed. These conditions represent the intentions and attitudes of the physiotherapists and patients who engaged in clinical interactions.^31^ However, no studies have examined the therapeutic alliance with regard to the specific setting (single vs. group) in rehabilitation.

Notably, scores for the Bond subscale of the WAIP in the single treatment groups were higher than in the group setting, supporting a better therapeutic alliance in individualized treatment. The exclusivity of the relationship between the patient and physiotherapist promotes a longer lasting bond of trust by better controlling the patient's experience with respect to the expectations of rehabilitation.

With regard to personality profile and the therapeutic alliance, in the total sample and group treatment, the Masculine/Feminine MMPI-2 subscale was negatively associated with therapeutic alliance, showing that women who feel discomfort in their female identity, independence, and self-confidence have difficulty in establishing an alliance with the physiotherapist.

It is conceivable that this attitude arises from the belief of being able to help themselves independently and the feeling of not needing external help. Furthermore, for the total sample and group treatment, greater psychological impairment correlated with less pain reduction from T0 to T1, particularly for women who were excessively worried about their state of health (Hypochondriasis MMPI-2) and somatic symptoms (Hysteria MMPI-2) and who were wary of others (Paranoid MMPI-2).

A recent study showed that high somatization scores were predictive of increases in sensorial, affective, and cognitive dimensions of clinical pain^32^ and that hysteria and hypochondria are present in chronic pain sufferers,^33^ highlighting the main role of the self-evaluation of somatic symptoms in the perception of pain. Regarding the paranoia subscale and its correlation with pain, it is conceivable that women with higher paranoia scores are more centered on themselves and their body sensations than to others, due to their distrust.

Overall, these results demonstrate that psychological impairments affect pain perception after physiatric treatment; analyzing the two treatment groups individually, we found that with only individual treatment was the tendency to hide problems of the emotional control (Correction MMPI-2) associated with less pain reduction at T1. Furthermore, in the single group, only defensive attitude (Lie MMPI-2) was negatively associated with the therapeutic alliance and any other psychopathological traits. Before starting any physiatric treatment, it is important to determine the level of psychopathology to direct patients to single or group treatment.

### Strengths

In rehabilitation, the single versus group setting has not been examined in breast cancer survivors; our study considered the psychoemotional factors of the patients and the therapeutic alliance.

### Weaknesses

We decided to analyze the profile and the relationship that the patient established with the physiotherapist without analyzing his personality profile, starting from the assumption that the physiotherapist was a healthy and psychologically balanced subject. Future studies will be needed to detail this aspect.

## Conclusion

Proposing the same rehabilitative intervention in both breast cancer groups, our results showed significant reduction in pain and good functional recovery of the upper limb, which did not depend on the setting (single or group). However, with single rehabilitation treatment, patients developed a better therapeutic alliance and experienced a more comfortable environment.

The therapeutic relationship between the patient and physiotherapist is a central component in breast cancer care and its functional recovery. Moreover, our data suggest that the single rehabilitative treatment setting establishes a better therapeutic alliance and favors improvements in emotional and physical function. Other studies should examine the therapeutic alliance in cancer patients more extensively by considering the psychoemotional aspects of rehabilitative treatment as much as the functional aspects.

## Abbreviations Used

BMI: body mass index
CONSORT: Consolidated Standards of Reporting Trials
DASH: Disabilities of Arm, Shoulder and Hand Questionnaire
MMPI-2: Minnesota Multiphasic Personality Inventory
VAS: visual analog scale
WAIP: Working Alliance Inventory Patient form
